# Yellow Fever at Savannah

**Published:** 1876-09

**Authors:** 


*Yellow Fever at Savannah.*—As our readers are aware, a fearful epidemic of yellow fever has appeared at Savannah, Ga., and is sweeping the coast of Georgia. In virulence and mortality, the epidemic at Savannah is unparalleled in the history o that city. The sanitary surroundings of Savannah have greatly contributed to the introduction and spread of the fever. The sewage of Savannah has been deposited close to the city, and in such a way as to contaminate the air and give intensity to the fever poison. We shall take occasion at some future time to criticise the defective sanitary arrangements of our sister city. We deeply sympathize with our distressed fellow-citizens in their awful calamity, and trust soon that the epidemic shall spend its force. The physicians of Savannah have labored only as true physicians can in the cause of suffering humanity. The people of that city owe them an eternal debt of gratitude. In the future, when the calm comes, they should ever remember the heroic sacrifices made by them for the lives of her stricken people.

